# Effectiveness of pneumococcal conjugate vaccination against invasive pneumococcal disease among children with and those without HIV infection: a systematic review and meta-analysis

**DOI:** 10.1186/s12879-019-4325-4

**Published:** 2019-08-05

**Authors:** Hossein Molavi Vardanjani, Hodjat Borna, Ali Ahmadi

**Affiliations:** 1 Molecular Biology Research Center, Systems Biology and Poisonings Institute, Baqiyatallah University of Medical Sciences, Tehran, Iran; 2 Chemical Injuries Research Center, Systems Biology and Poisonings Institute, Baqiyatallah University of Medical Sciences, Tehran, Iran

**Keywords:** Invasive pneumococcal disease, HIV, Children, Pneumococcal conjugate vaccine, Treatment outcome

## Abstract

**Background:**

HIV-infected children are at a higher risk of Invasive Pneumococcal Disease (IPD) and its mortality, even in the era of antiretroviral therapy. Therefore, an effective vaccination strategy would be beneficial. To investigate the effectiveness of Pneumococcal Conjugate Vaccination (PCV) against IPD among HIV-Infected and HIV-Uninfected Children through a systematic review and meta-analysis.

**Methods:**

Observational studies and randomized trials on 7 years old or older children were searched in the Cochrane Library, Web of Science core collection, Embase, Medline/PubMed, and Google Scholar. Critical appraisal was done using the Cochrane risk of bias tool and the Newcastle-Ottawa quality assessment form. Effectiveness and efficacy of at least one dose of PCV was investigated among children with and without HIV considering subgroups of pneumococcal serotypes. We meta-analyzed the effect sizes using random-effects modeling.

**Results:**

Efficacy of PCV was estimated as 45.0% (31.2, 56.1) and 52.6% (25.7, 69.8) among HIV-infected and HIV-uninfected children, respectively. Effectiveness of PCV among HIV-infected children as − 6.2% (− 67.6, 32.7) was significantly lower than HIV-uninfected children 65.1% (47.3, 76.9). Effectiveness of PCV among HIV-infected children for IPDs caused by vaccine serotypes was estimated as 7.7(− 66.7, 48.9), and for IPDs caused by non-vaccine serotypes was estimated as − 402.8(− 1856, − 29.2).

**Conclusion:**

Unlike the evidence on the efficacy of PCV against IPD among both of HIV-infected and HIV-uninfected children, its effectiveness against IPD among HIV-infected children is much less limited.

**Review registration:**

The study protocol was registered at PROSPERO (registration ID: CRD42018108187).

**Electronic supplementary material:**

The online version of this article (10.1186/s12879-019-4325-4) contains supplementary material, which is available to authorized users.

## Background

Children with HIV-infection (HI) are prone to invasive pneumococcal disease (IPD) roughly 40 times more than those without HIV-infection (HUI) [1, 2]. The risk of IPD-related mortality among them is higher than children without HIV-infection (HUI) [3, 4]. Prevention of IPD incidence is the most effective strategy to reduce the burden of IPD among children with HIV-infection (HI).

Of the most appropriate preventive strategies, for the time being, may be boosting the immune system function through antiretroviral therapy (ART) and/or vaccination against pneumococcal diseases [2]. Nevertheless, even after adopting ART, risk of incidence of IPD among children with HIV-infection (HI) remains high [5–7]. Therefore, effective vaccination can prove to be an important strategy for prevention of IPD and IPD-related mortality in HI [8, 9].

Currently, pneumococcal polysaccharide vaccines (PPVs) and pneumococcal conjugate vaccines (PCVs) are being used as effective immunizations against pneumococcal diseases in the general population [7, 8, 10, 11]. PCVs were followed PPVs and formulized to compensate for the defective induced immunization by PPVs in younger children, via stimulation of T-cell dependent immune system and boosting anamnestic responses [12–15]. In 2000, PCV7 was licensed, and when some evidence showed replacement of non-vaccine serotypes, the prevalent replaced serotypes were added to the vaccine and PCV13 was formulated and licensed by 2010 [16, 17].

Although PCV (i.e. PCV13 and PCV7) are currently recommended for use in children under 2 years old, studies have demonstrated mixed reports including lower effectiveness of these vaccines against IPD in HI children [5, 18–20].

On the other hand, the recent review [21], though conducted by experienced specialists in the field, did not adopt a systematic approach for search and review; a defect which may results in biased findings. What is more, the existing pertinent reviews [21, 22] have mainly focused on the findings of well-known randomized trials, which may cause the overestimation of the vaccines’ effectiveness or biased findings, as an outcome of ignoring small studies and/or observational studies.

In this systematic review and meta-analysis of randomized trials and observational studies, we aimed at studying the efficacy and effectiveness of PCVs against IPD among children (younger than 7 years old) with and those without HIV-infection.

## Methods

This systematic review and meta-analysis is reported in accordance with the Referred Reporting Items for Systematic Reviews and Meta-Analyses (PRISMA) statement [23]. The study was registered at the International Prospective Register of Systematic Reviews (registration ID: CRD42018108187).

The Cochrane Library, Web of Science core collection, Embase, Medline/PubMed, and Google Scholar were searched to identify English language reports published up to April 2018 (without any date restriction).

Appropriate combinations of pre-specified words, including “community acquired”, “invasive pneumococcal disease”, “pneumonia”, “infection”, “streptococcus”, “bacteremia”, “meningitis”, “bacterial”, “sepsis”, “conjugate vaccine”, “PCV”, “Prevnar”, “Prevenar”, “PHiD-CV”, “immunization”, “vaccination”, “protection”, “effectiveness”, “efficacy”, “effect”, “mortality”, “impact”, “benefit”, “prevention”, “control”, “epidemiology”, “hospitalization”, “morbidity”, and “incidence” were used. The bibliographies of reviews and other publications fully related to pneumococcal diseases were checked for additional reports.

Pre-post studies, time series, multiple interrupted time series, case-control studies, randomized trials, indirect cohort studies, case-coverage, case-cohort studies, and laboratory-based or population-based surveillance studies were eligible for inclusion into the study. Studies comparing vaccinated children to unvaccinated children in terms of IPD incidence (risk), or studies comparing children with and without IPD in terms of odds of any previous vaccination with PCVs were eligible for inclusion.

The issue of searching articles and their primary screening were conducted regardless of considering HIV infection status in participants, since some of the studies might have separately reported their results according to HIV status as study subgroups. Although our target population was children younger than 7 years old, to retrieve any article included this age group even as a subgroup in the text body (but not in the title or abstract). No age restriction was imposed during online searches.

Two primary outcomes of the study are the effectiveness and efficacy of PCVs for the prevention of IPD (i.e. all serotypes (AT IPD)) including meningitis, sepsis, and bacteremia. Our secondary outcomes were effectiveness and efficacy of PCV against non-vaccine serotypes (NVT IPD), and vaccine serotypes (VT IPD) IPDs, separately. Vaccine serotype IPD (VT IPD) indicates the infection by any of the PCV7, PCV9, PCV10, and PCV13 included serotypes.

Titles and abstracts of the publications were screened by two independent reviews to identify eligible publications. Then, full text of eligible publications identified in the previous steps as reviewed independently. Finally, only eligible studies that had reported their results separately by HIV status (i.e. HIV infected and/or HIV uninfected), were included in the critical appraisal phase of the study. The Cochrane risk of bias tool for randomized trials [24] and the most appropriate Newcastle-Ottawa quality assessment Form for nonrandomized studies [25] were used to assess the risk of bias. Appraised publications were categorized into three risk of bias groups, including high, unclear, and low risk, according to their obtained scores in critical appraisal.

A form was designed for data collection. According to a comprehensive literature review, a list of potentially correlated variables with the effectiveness and/or efficacy of vaccination in children as well as several methodological factors that may had some insidious effects on the results of the studies was provided. Then data collection form was provided accordingly. Afterwards, data were extracted into the data form. Disagreements over the screening and critical appraisal as well as extraction of data were discussed in the team until consensus was reached.

### Data analyses

We pooled effect measures (i.e. relative risk (RR) or odds ratio (OR)) by subgroups of causative pneumococcal serotypes, study design, and HIV status (infected, and uninfected) by using Random-Effects model if meta-analysis was applicable. The DerSimonian and Larid method was applied.

Considering the well-evidenced issue of the probability of serotype replacement after the PCV vaccination, and also to show if the efficacy and effectiveness of the PCV vaccination is depended on the IPDs’ causative serotypes, meta-analyses were done in three subgroups including IPDs caused by all serotypes (AT IPD, showing the overall results of the PCV vaccination); IPDs caused by vaccine-serotypes (VT IPD, showing the results of the PCV vaccination only against serotypes which are included in the PCVs; and IPDs caused by the non-vaccine serotypes (NVT IPD, showing the results of the PCV vaccination against IPDs which their causative serotypes are not included in the PCVs, in this case if the efficacy or effectiveness be a negative value it may be an evidence of serotype replacement).

In addition, as the results reported by the RCTs show estimates of the vaccine efficacy, while reported results by the observational study show estimates of vaccine effectiveness, all serotype-based subgroup meta-analyses were repeated according to the subgroups defined based on the study design (pre-post, case-control, and RCT).

The percentage of efficacy or effectiveness and their 95% confidence interval were calculated using following formula:

$$ \mathrm{Effect}\mathrm{iveness}\%=\left(1-\mathrm{Pooled}\ \mathrm{Effect}\ \mathrm{measure}\right)\times 100 $$

Where pooled effect measure was “pooled RR” or “pooled OR” according to the study design.

If an individual study had reported RR/OR for different subgroups (e.g. for subgroups by causative serotypes of IPD), its subgroup specific RR/ORs considered independent and were pooled by using Random-Effects model.

As in the meta-analysis studies a crucial criterion for doing meta-analysis is a relative homogeneity of the observed primary effect measures (in this study efficacy and/ or effectiveness), we assess homogeneity of the primary effect measures. In a simplified word, homogeneity means that the sampling population of each of the included primary studies is adequately similar with the sampling population of the other studies. Meta-analysis of effect measures from studies with different sampling populations results in an apple-orange juice. The Q test and the I^2^ test were used to assess heterogeneity of individual estimates. In the cases of high heterogeneity, meta-regression models were fitted to identify variables affecting this heterogeneity by using restricted maximum likelihood technique. Meta-regression is a technique to identify determinants of differences between the sampling population of the primary studies (i.e. heterogeneity).

L’Abbe and Galbraith plots were used to identify influential studies. Egger and Funnel plot tests were used to evaluate for publication bias in each study subgroups. We also used sensitivity analysis to evaluate the effect of each individual estimates on the pooled effect measure. The data were analyzed using Stata version 11.2 (Stata Corporation, College Station, TX, USA).

## Results

### Overview of included studies and participants

**Fig. 1:**
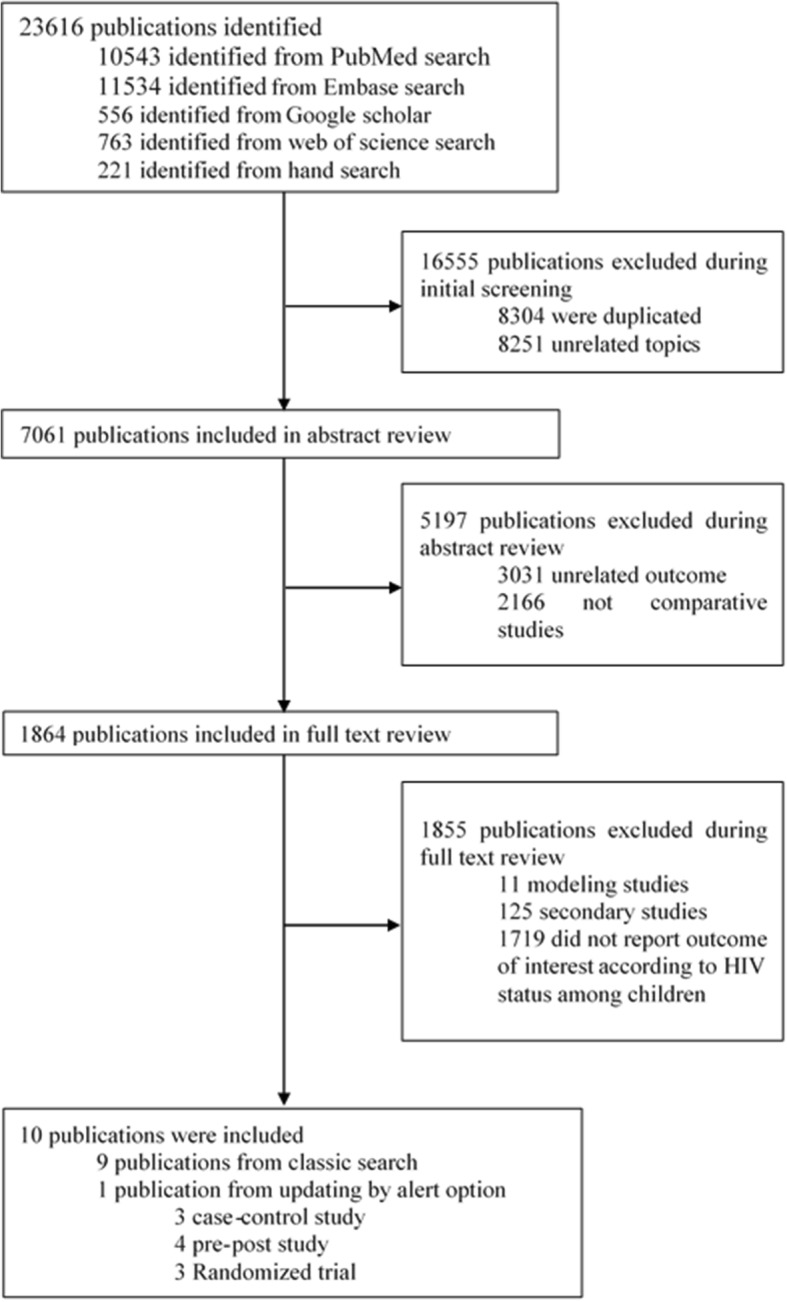
Study selection flow diagram

A total of 23,616 articles identified, of which 1864 were screened to be included in the full text review (Fig. 1).

Ten studies were included in the study. Out of these, one was conducted in the USA and nine studies in the South Africa. No cohort study was identified. The analyzed data includes 1332 HI and 3462 HUI children in three case-control studies; 2577 HI and 37,259 HUI children in three randomized trials; and 48,550 HI and 2,272,443 HUI children in four pre-post studies (Table 1).

**Table 1:** Publications included in meta-analysis

No.	Author	HIV infe.	Vaccine	Outcome	Age group	Schedule	Study design	Sample size	Risk of bias
1	Klugman et al. [20]	HI, HUI	PCV9	First episode of IPD: All serotype, vaccine and non-vaccine serotypes; during 2.3 years of follow up	Less than 5 years	3 doses at 6, 10, 14 WK; no booster dose	Randomized trial	39,836	Low
2	Madhi et al. [26]	HI, HUI	PCV9	First episode of IPD: All, vaccine and non-vaccine serotypes; during 6.16 years of follow up	Less than 7 years	3 doses at 6, 10, 14 WK; no booster dose	Randomized trial	39,836	Low
3	Madhi et al. [27]	HI, HUI	PCV9	Bacteremic pneumococcal pneumonia^a^: all and vaccine serotypes; during 2.3 years of follow up	Less than 5 years	3 doses at 6, 10, 14 WK; no booster dose	Randomized trial	39,836	Low
4	Cohen et al. [19]	HI, HUI	PCV7	IPD: All and PCV7 serotypes	Less than 1 year	2 + 1 at 6 and 14 WK, and 1 at 9 month (two or more)	Matched case control	1395	Low
5	Cohen et al. [18]	HI, HUI	PCV13	IPD: All, PCV7 and PCV13 serotypes	Less than 5 years	2 + 1 at 6 and 14 WK, and 1 at 9 months (two or more)	Matched case control	1716	Low
6	Mollendorf et al. [28]	HUI^a^	PCV7	IPD: vaccine serotypes^a^	Less than 5 years	2 + 1 at 6 and 14 WK, and 1 at 9 months (two or more)	Matched case control		low
7	Steenhoff et al. [29]	HI	PCV7	IPD: vaccine and non-vaccine serotypes	Less than 5 years^a^	3 + 1 at 2, 4, 6 and 12–15 months	Retrospective Pre-post	256	unclear
8	Nzenze et al. [30]	HI, HUI	PCV7, PCV13	IPD: All, PCV7, PCV13 and non-vaccine serotypes	Less than 2 and 2–5 years	2 + 1 at 6 and 14 WK, and 1 at 9 months	Pre-post	160,000	unclear
9	Gottberg et al. [5]	HI, HUI	PCV7, PCV13	IPD: All, PCV7, PCV13 and non-vaccine serotypes	Less than 2 years	2 + 1 at 6 and 14 WK, and 1 at 9 months	National Pre-post	≈2,000,000	Unclear
10	Mollendorf et al. [4]	HI, HUI	PCV13	IPD: All serotypes	Less than 1 year	2 + 1 at 6 and 14 WK, and 1 at 9 months	Pre-post	937^a^	Unclear

*Abbreviations: WK* Weeks, *HI* HIV-infected, *HUI* HIV-uninfected, *IPD* invasive pneumococcal disease, *PCV* pneumococcal conjugate vaccine

^a^Due to methodological considerations or unavailability of primary information only this part of results was included in the meta-analysis

### Overall and subgroup-specific pooled estimates of efficacy/effectiveness

**Fig. 2:**
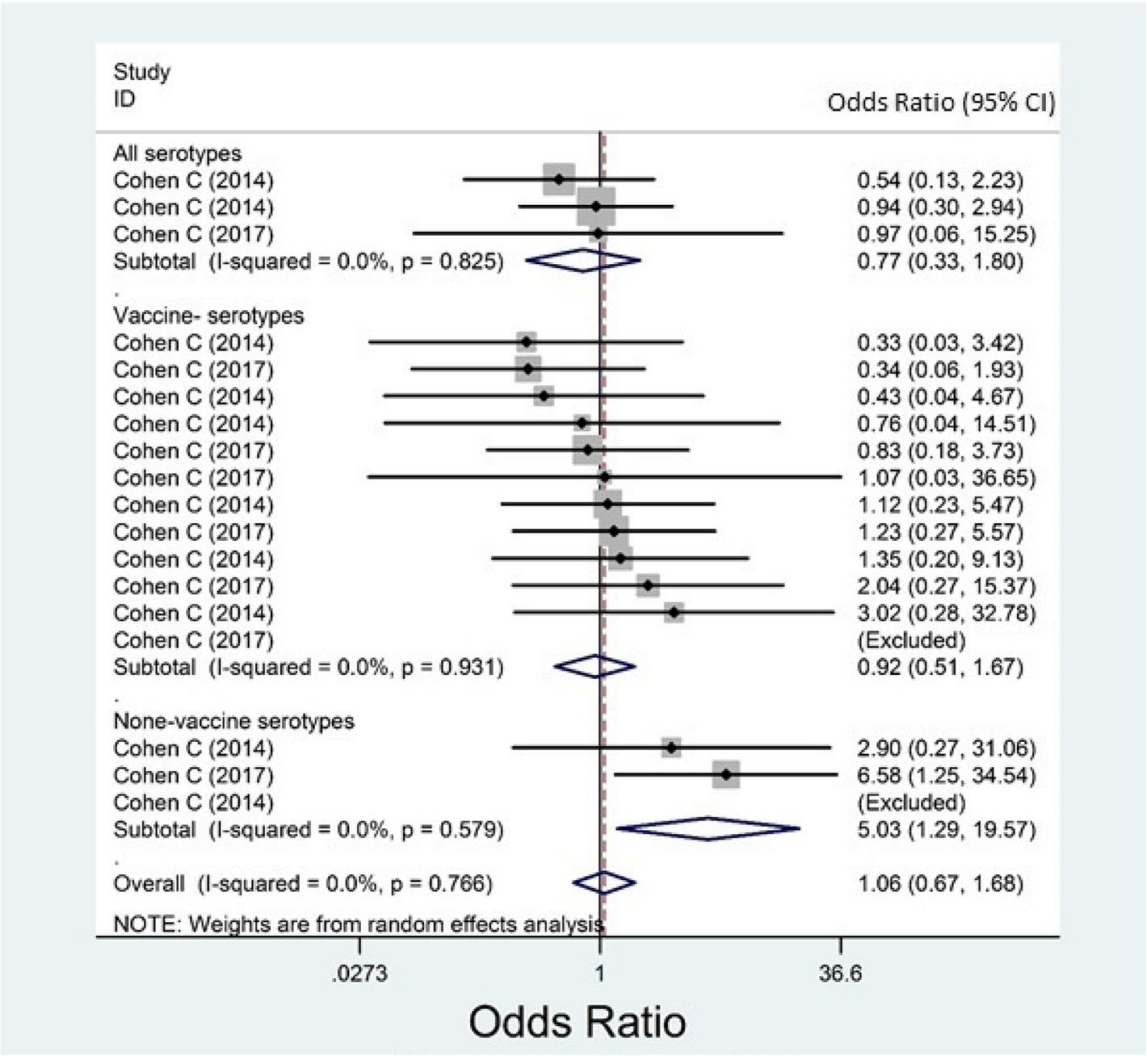
Forest plot of the effectiveness (VE %) of pneumococcal conjugate vaccines against invasive pneumococcal disease among HIV-infected children according to case-control studies

**Fig. 3:**
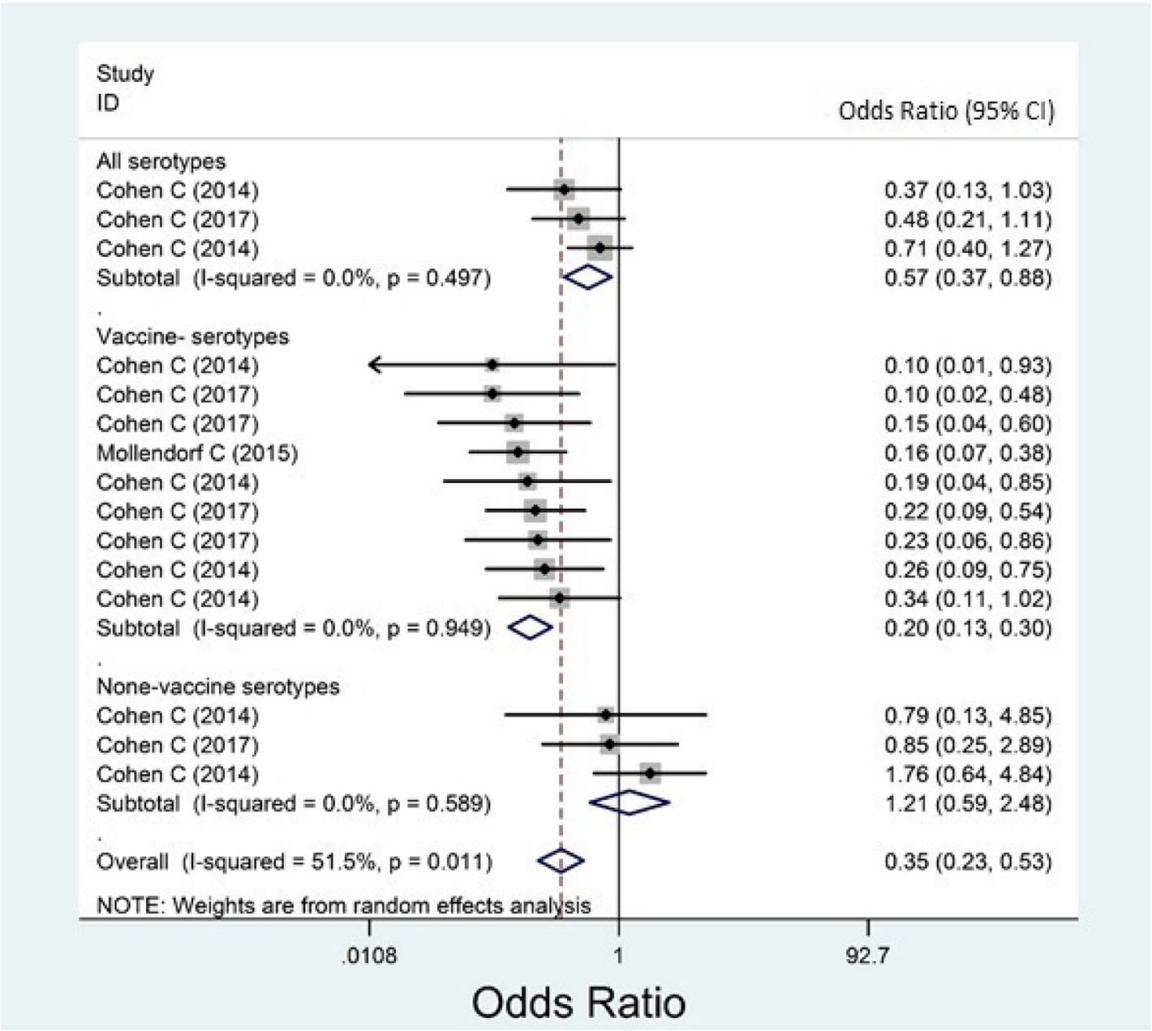
Forest plot of the effectiveness (VE %) of pneumococcal conjugate vaccines against invasive pneumococcal disease among HIV-uninfected children according to case-control studies

**Fig. 4:**
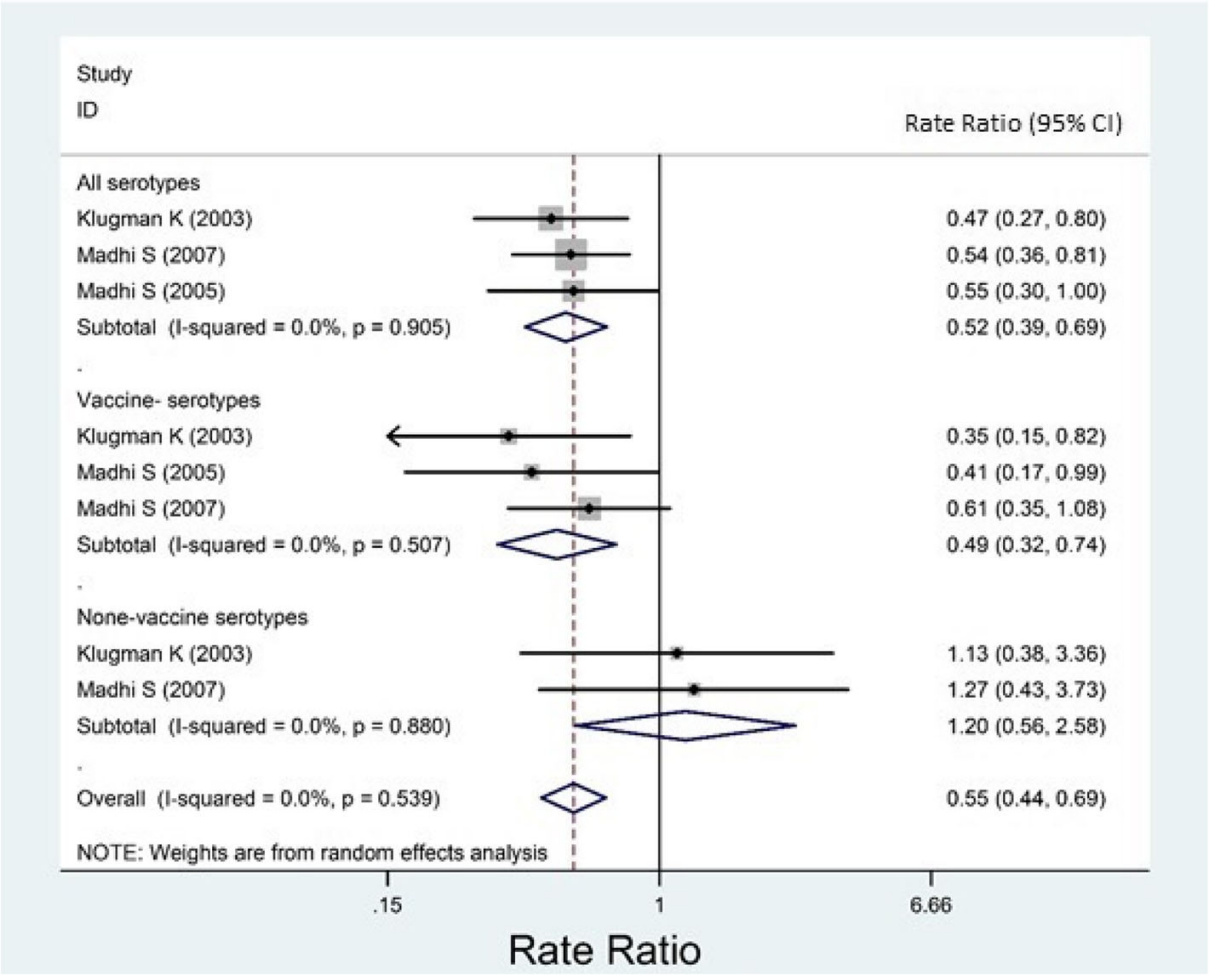
Forest plot of the efficacy (% VE) of pneumococcal conjugate vaccine against invasive pneumococcal disease among HIV-infected children according to randomized trials

**Fig. 5:**
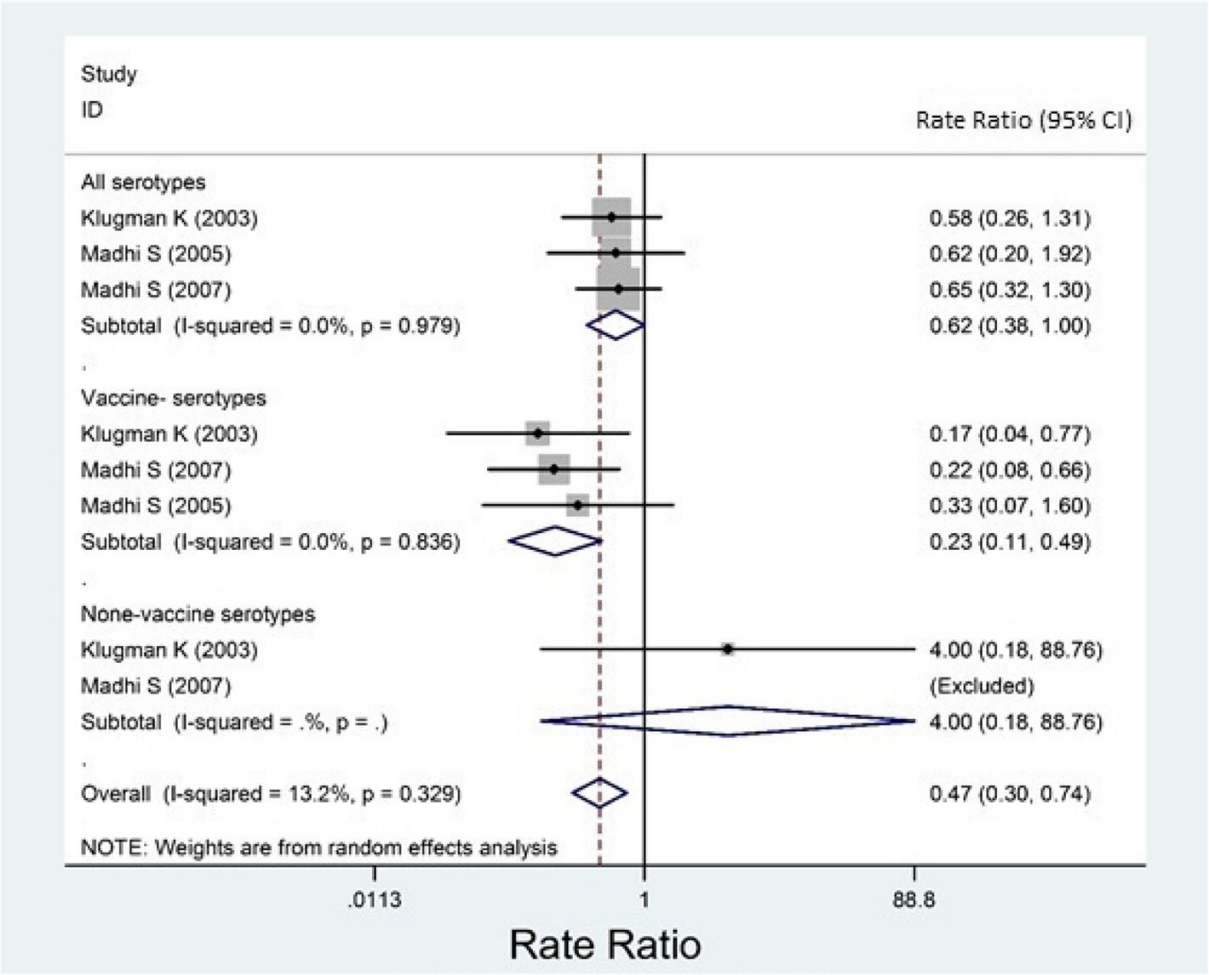
Forest plot of the efficacy (% VE) of pneumococcal conjugate vaccine against invasive pneumococcal disease among HIV-uninfected children according to randomized trials

Almost all of the included studies had reported their results for overall IPD, but not for meningitis, sepsis, or bacteremia, separately. Based on case-control studies, the overall effectiveness of PCVs against any IPD was estimated as − 6.2% (95% CI: − 67.6, 32.7, Fig. 2) and 65.1% (95% CI: 47.3, 76.9, Fig. 3) among HI and HUI children, respectively. Based on randomized trials, the overall efficacy among HI and HUI children was estimated 45.0% (31.2, 56.1, Fig. 4) and 52.6% (25.7, 69.8, Fig. 5), respectively.

Because of high heterogeneity, we are unable to meta-analyze the individual estimates reported by pre-post studies, however, meta- analysis was done in some subgroups with moderate heterogeneity (Table 2).

**Table 2:** Effectiveness or efficacy (95% CI) of PCVs against invasive pneumococcal disease (IPD) according to different study designs, among HIV-infected and HIV-uninfected children

Pneumococcal serotypes	HIV status	Study design		
		Randomized trial	Case-control	Pre-post
All types	HI	47.9 (30.7,60.9)	22.6(−80.0, 66.8)	NA
	HUI	38.0(−0.1, 61.7)	43.0 (12.2, 63.0)	NA
Vaccine types	HI	51.0 (25.9, 67.7)	7.7(−66.7, 48.9)	NA
	HUI	77.3 (50.9, 89.5)	80.0 (70.4, 86.5)	NA
Non-vaccine types	HI	−20.0(− 158, 44.2)	−402.8(−1856, −29.2)^a^	21.3(−9.5, 43.5)
	HUI	− 300(− 8776, 82.0)	−21.1(− 147.8, 40.9)	−12.5(−46.5,13.6)^b^

*NA* Meta-analysis was not applicable due to high heterogeneity, *HI* HIV-infected, *HUI* HIV-uninfected

^a^For children younger than 2.5 years

^b^For PCV7 and PCV13, but not for other vaccines

The heterogeneity of individual studies was also high in subgroups, especially in pre-post studies.

### Determinants of heterogeneity of results by subgroups

The I^2^ index, for pre-post results, was 85.2% with a *P*-value of < 0.001 for AT IPDs among HI children (showing that meta-analysis is not justifiable in this subgroup), 75.4% (*P* < 0.001) for VT IPDs (showing that meta-analysis is not justifiable in this subgroup), and 15.5% (*P* = 0.316, showing that meta-analysis is justifiable in this subgroup) for NVT IPDs (Additional file 1: Figure S1).

The age group (*P* = 0.019), the vaccine valency (*P* = 0.012), the vaccination schedule (*P* = 0.002), the time after the introduction of the vaccination program in the population (*P* = 0.027), the percentage of children received a booster dose (*P* = 0.05), and the IPD diagnostic method (*P* = 0.04) involved in this heterogeneity (factors which may be the underlying causes of different sampling populations of the included studies)). These factors were quite different depending on the type of outcome and the HIV-infection status.

Among HUI children, the I^2^ index for heterogeneity of the pre-post studies was estimated as 93.7% (*P* < 0.001) for AT IPD, 92.3% (*P* < 0.001) for VT IPD, and 66.9% (*P* = 0.006) for NVT IPD (Additional file 1: Figure S2). The percentage of the population who had received a booster dose (*P* = 0.004), the vaccination schedule (*P* = 0.012), the time passed from the beginning of the vaccination program (*P* = 0.011), and the vaccine valency (*P* = 0.047) were the effective factors on the heterogeneity of results in HUI children.

The I^2^ index for the case-control studies was estimated as 0.0% (*P* = 0.825); 11.7% (*P* = 0.931); and 0.0% (*P* = 0.579) for AT, VT and NVT IPDs among HI children, respectively (Fig. 2, showing that meta-analysis is justifiable in each of these subgroups).

The I^2^ index, of case-control results for HUI children, was estimated as 0.0% (*P* = 0.497); 0.0% (*P* = 0.949); and 0.0% (*P* = 0.589), respectively for AT, VT and NVT IPDs (Fig. 3, again as above).

The vaccination schedule (*P* = 0.039), the vaccine valency (*P* = 0.005), and the percentage of children who received a booster dose (*P* = 0.042), were the effective factors on the heterogeneity of results in the case-control studies.

The I^2^ index for reported results of randomized trials was low and estimated as 0.0% (*P* = 0.539), and 0.13.2% (*P* = 0.329) among HI and HUI children, respectively (Figs. 4 and 5).

### Age subgroups

In pre-post studies, because of the high heterogeneity of results in age subgroups both in HI and HUI children, we did not meta-analysis (Additional file 1: Figure S3 and Figure S4).

Although the results of the case-control studies were not heterogeneous among HI children, they involved only the age group younger than 2.5 (VE % = − 6.2; I^2^ = 0.0%; Fig. 2). The results of these studies had moderate heterogeneity for HUI children (I^2^ = 51.5%; *P* = 0.011). The effectiveness of the PCV was estimated by age-group (as a determinant of vaccine efficacy/ effectiveness) as follow: 84.0% (62.0, 93.0) for children younger than 5 and 62.2% (42.7, 75.0) for children younger than 2.5 (Additional file 1: Figure S5 and Figure S6).

According to the randomized trials, the efficacy of the PCV in HI children was 48.7% (11.9, 70.1), 49.9% (17.8, 69.4) and 39.1% (14.8, 56.4), for children younger than 2.5, 5, and 7 years old, respectively (Additional file 1: Figure S7). In addition, these values for HUI children were 51.0% (− 67.0, 86.0), 50.0% (− 25.5, 80.0), and 58.7% (− 16.8, 85.4), respectively (Additional file 1: Figure S8).

### Vaccine valency subgroups

Due to high heterogeneity, conducting a meta-analysis in pre-post studies was not applicable based on the vaccine valency (Additional file 1: Figure S9 and Figure S10). The effectiveness was estimated in the case-control studies (Table 3, and Additional file 1: Figure S11 & Figure S12) by vaccine valency (vaccine valency is an important determinant of the PCVs efficacy and/ or effectiveness, as the preventive effect of typeable PCVs is a function of the serotypes added in the vaccine). Randomized trials had only reported the efficacy of PCV9 (Figs. 4 and 5).

**Table 3:** Effectiveness (95% CI) of pneumococcal conjugate vaccine according to the case-control studies by HIV infection status and vaccine valency

HIV status	PCV^a^	PCV7	PCV13	Overall
HI	10.4(−100.1, 60.0)	7.8(−68.2, 49.5)	−251(− 1944, 40.0)	−6.2% (− 67.6, 32.7)
HUI	80.7 (47.3, 90.1)	60.5 (30.0, 77.7) ^b^	57.8 (0.5, 82.1)	65.1% (47.3, 76.9)

*HI* HIV-infected, *HUI* HIV-uninfected, *PCV* pneumococcal conjugate vaccine

^a^ Unspecified vaccine valency in primary studies

^b^ Meta-analysis was done despite of moderate heterogeneity (I-squared around 50%); more details in Additional file 1

### Sensitivity analysis and assessment of publication bias

The results of sensitivity analysis showed that despite of some variations in individual estimates, none was influential (Additional file 1: Figures S13–18). As there was no study with poor appraisal score, no sensitivity analysis was done with regard of the quality of publications. The results of funnel plot and Egger’s test for publication bias in randomized trials (*P*-value _HI_ = 0.358, *P*-value _HUI_ = 0.839, Additional file 1: Figures S19–S20), case-control studies (P_HI_ = 0.210, P_HUI_ = 0.419, Additional file 1: Figures S21–S22), and pre-post studies (P_HI_ = 0.192, P_HUI_ = 0.254, Additional file 1: Figures S23–S24) showed a lack of publication bias.

## Discussion

In this systematic review and meta-analysis, we pooled the available data on the efficacy and effectiveness of PCVs against IPD among HI and HUI children, as well as correlates of heterogeneity of the effect of PCV to prevent IPD. The results showed that the overall effectiveness (efficacy in the real situation) of PCVs in preventing IPD (including both of vaccine or non-vaccine serotype IPDs) among HI children was significantly lower than HUI children (− 6.2% vs. 65.1%). It may be due to higher rates of NVT IPD in HI children compared to HUI children (VE for NVT: in HI, − 403% VS in HUI, − 21%). However, the pooled efficacy among children with and without HIV was not significantly different (45.0% vs. 52.6%).

The higher efficacy by randomized trials compared to observational studies in a non-differential direction, is an expected and documented issue [17, 31]. However, the differences observed in our study results was differential and reverse, as the effectiveness of vaccination among HI children was 52% less than its efficacy (− 6.2 of effectiveness VS 45% of efficacy), but 12% more than efficacy among HUI children (65% of effectiveness VS 53% of efficacy). The higher PCV efficacy (compared with its effectiveness) in HI children might be interpretable as a result of the more available cares for participants of randomized trials (such as higher percentage of receiving ART, higher rate of vaccination, and also timeliness vaccination) compared to real population. Accordingly, improvement in the accessibility and utilization of the routine healthcare available for HI children in addition of extending the range of such services may be measures to increase the PCV effectiveness among this population.

Higher PCV effectiveness against VT IPD among HUI children might be due to indirect effects of other vaccination programs (such as vaccination with PPV23 or Influenza vaccine), and/or also induced herd immunity by PCV vaccination, explaining the 7.7% effectiveness against VT IPDs among HI children compared to 51% in HUI children [32–35].

The difference between the effectiveness and efficacy of PCVs in HI children was − 25% against AT IPD, − 383% against NVT IPD, and only − 43% against VT IPD. This is while, surprisingly in HUI children, the effectiveness of PCVs against NVT IPD was improved up to about 280% compared with the efficacy. This major difference might be due to a serious situation – for example, replacement of vaccine-serotypes and, especially non-vaccine serotypes in HI children happens faster and more than in HUI children [36–38]. The pattern of replaced serotypes in HI children would be so important since vaccination may operate reversely on these children, and higher-valency vaccines or vaccines with high level of cross-serotype protection might be needed after a short period of time [39, 40]. This probable serotype replacement may be a result of a type of false assurance after vaccination, and then a reduction in the healthcare provided for HI children, and consequently more exposure with NVT serotypes. Therefore, it is necessary to healthcare provider and care-givers of HI children be aware about this type of false assurance.

Although we tried to study children under 7 years of age, which was referred to as a homogeneous age group in previous studies [41, 42], our results showed that the age group can affect the efficacy and effectiveness of vaccines in both HI and HUI children. Although the study results showed that older HI children had the lowest efficacy compared to children under 2 years old, this result may be due to the length of follow-up time (around 6 years VS less than 2 years) and is not an follow-up adjusted valuable result [26]. However, it may be an indicating evidence on the need of booster doses to increase the vaccine effectiveness among older children.

The results of this study also showed that vaccination schedule could have an effect on vaccine’s effectiveness. This finding is in agreement with the results of previous studies about the necessity of booster dose in the vaccination program for pneumococcal diseases [43–45]. Besides, the valency of vaccine used in the vaccination program caused heterogeneity in effectiveness, especially among HI children, as these values for PCV7 and PCV13 were 7.8% and − 251%, respectively. At the same time, PCV13 contains serotypes which were replaced a few years after the PCV7 vaccination in routine vaccination programs [46, 47]. Higher effectiveness of PCV7 may be due to higher herd immunity that is expected as time pass from the introduction of the PCV7 vaccination program [16, 33]. The lower value of effectiveness for PCV13 compared with PCV7, which only belongs to HI children, could be a further reason on the faster and higher replacement of non-vaccine pneumococcal serotypes among HI children. Accordingly, it seems that increase in the valency of the PCVs may not be a single effective measure to prevent the IPDs and it should be belong with other interventions.

Another leading determinant of heterogeneity in observational studies was a methodological factor – the IPD diagnosis method. As Madhi et al. [27] has shown the specificity and sensitivity of various methods for the diagnosis of pneumonia are so different [48, 49]. Therefore, it is necessary to separate the methods appropriate for therapeutic purposes from those suitable for public health goals.

Several randomized trials have been conducted to determine the impact of the PCV7 and PCV13 in HI and HUI children, but these studies have mainly been focused on the quality and quantity of host immune system responses to the vaccines, and not to the incidence rate of IPD among vaccinated children [15, 50–52]. Therefore, the available effect measures are limited to the PCV9.

We were unable to subgroup analysis for strata by dose of vaccine and vaccination schedule on the effectiveness of vaccination due to inadequacy of the number of individual studies. In addition, although we searched for data on the effectiveness of PCV against types of IPD (i.e. meningitis, sepsis, and bacteremia) no individual data was available in this regard.

We could not find any evidence of influential study and/or publication bias based on the sensitivity analysis and Egger’s test for publication bias. Accordingly, the results of the study are generalizable. As all studies except for one were conducted in the South Africa, the results of the study might be challengeable to be used in other countries; as a matter of course, it was because of the limitation of existing evidence.

## Conclusion

Effectiveness of PCV against IPD was at − 6.2 and 65.1% in HIV-infected, and -uninfected children, respectively. Its effectiveness against VT IPD among HI children was much less than its efficacy. Its effectiveness among HUI children was greater than its efficacy and it probably indicates the herd immunity of the PCV for HUI children but not for HI children. Not only does the vaccine not prevent IPD among HI children, it can lead to a rapid and high increase of vaccine-serotype and non-vaccine serotype cases in these children, either. The effectiveness of PCV13 among HI children was estimated as − 250% against all pneumococcal serotypes IPD.

## Additional file

Additional file 1: Results of subgroup analysis, in addition of publication bias and influential observation diagnostics. (DOCX 508 kb)

## Data Availability

The datasets used and analyzed during the current study are available from the corresponding author on reasonable request.

## Abbreviations

ART: Antiretroviral Therapy
AT IPD: All Serotypes
HI: Children with HIV infection
HUI: Children Without HIV Infection
IPD): Invasive Pneumococcal Disease
OR: Odds Ratio
PCV: Pneumococcal Conjugate Vaccination
PPVs: Pneumococcal Polysaccharide Vaccines
PRISMA: Reporting Items for Systematic Reviews and Meta-Analyses
RR: Relative Risk
VE %: Effectiveness
VT IPD: Vaccine Serotype IPD
VT IPD: Vaccine Serotypes
