# Supplementary material for: Effectiveness of pneumococcal conjugate vaccination against invasive pneumococcal disease among children with and those without HIV infection: a systematic review and meta-analysis
Source: BMC Infect Dis. 2019 Aug 5;19:685. doi: 10.1186/s12879-019-4325-4 (PMC6683423; doi:10.1186/s12879-019-4325-4)
Supplement: Supplementary file 1 — Results of subgroup analysis, in addition of publication bias and influential observation diagnostics. (DOCX 508 kb) [file 12879_2019_4325_MOESM1_ESM.docx]

## Additional file 1

**Figure S1: Forest plot of the Effect of PCV against IPD among HIV-infected Children based on Pre-post studies**


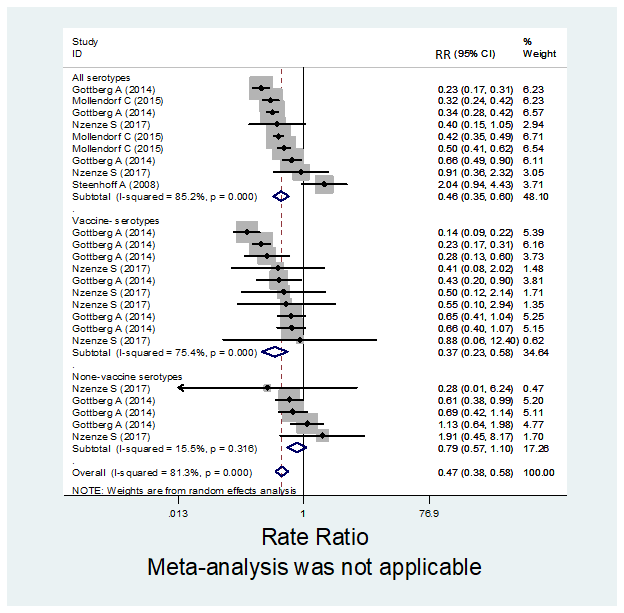


**Figure S2: Forest plot of the Effect of PCV against IPD among HIV-uninfected Children based on Pre-post studies**


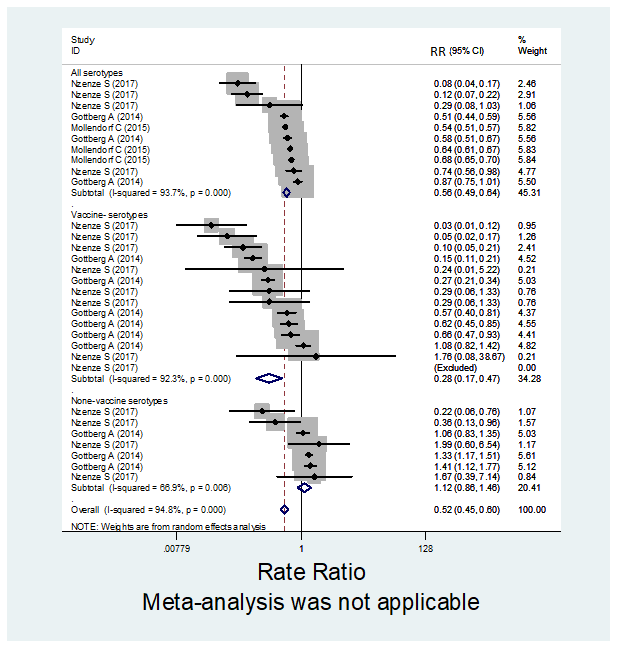


**Figure S3: Forest plot of the Effect of PCV against IPD among HIV-infected Children in pre-post studies according to age groups**


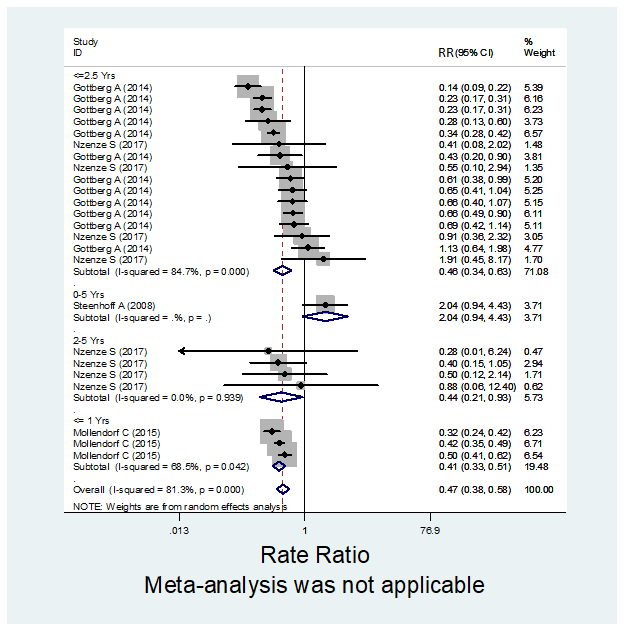


**Figure S4: Forest plot of the Effect of PCV against IPD among HIV-uninfected Children in pre-post studies according to age groups**

**
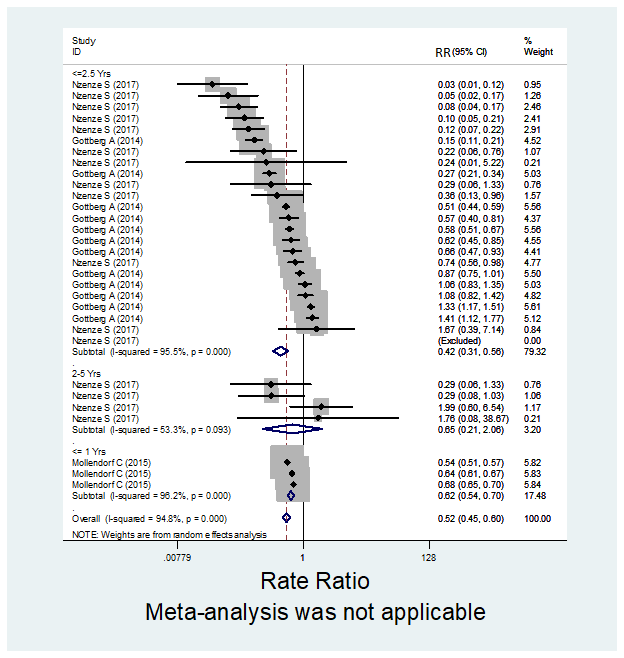
**

**Figure S5: Forest plot of the Effect of PCV against IPD among HIV-infected Children in case-control studies according to age groups**

**
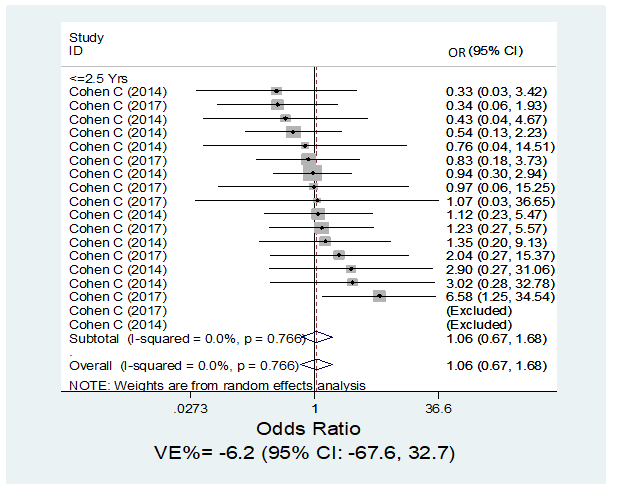
**

**Figure S6: Forest plot of the Effect of PCV against IPD among HIV-uninfected Children in case-control studies according to age groups**

**
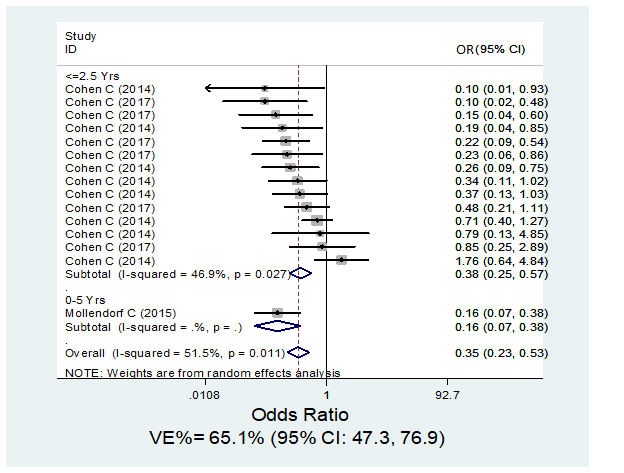
**

**Figure S7: Forest plot of the Effect of PCV against IPD among HIV-infected Children in randomized trials according to age groups**

**
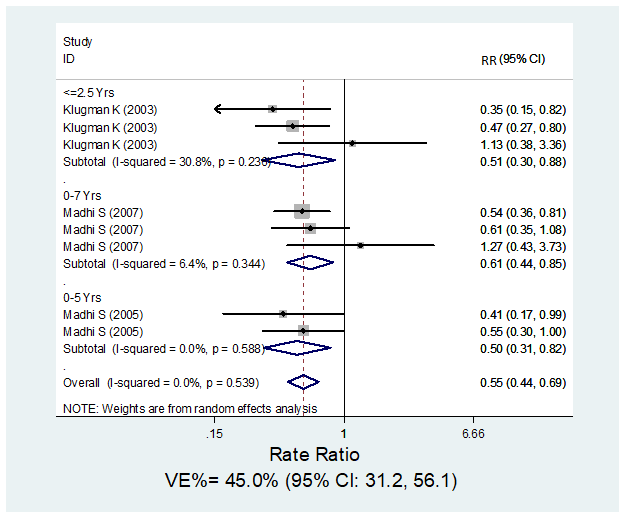
**

**Figure S8: Forest plot of the Effect of PCV against IPD among HIV-uninfected Children in randomized trials according to age groups**

**
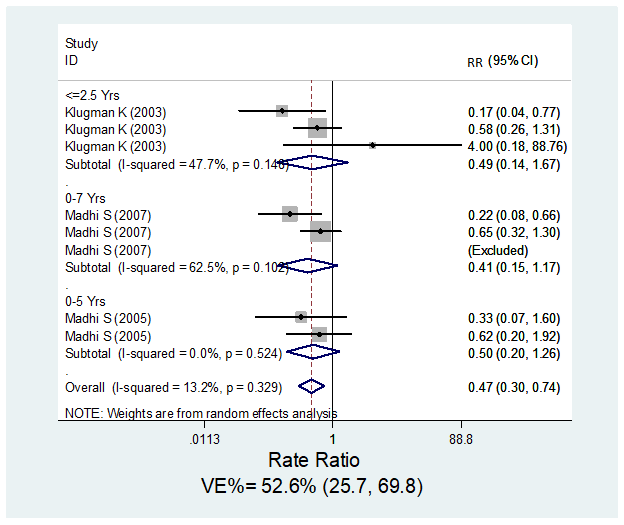
**

**Figure S9: Forest plot of the Effect of PCV against IPD among HIV-infected Children in pre-post studies according to vaccine valency**

**
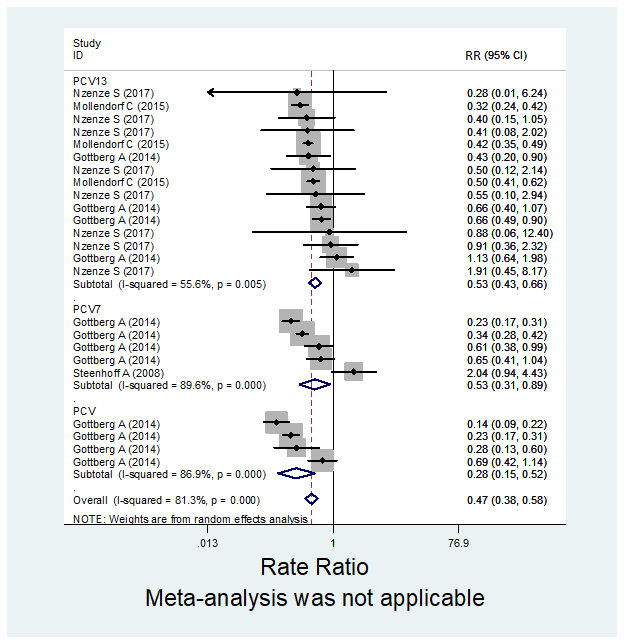
**

**Figure S10: Forest plot of the Effect of PCV against IPD among HIV-uninfected Children in pre-post studies according to vaccine valency**

**
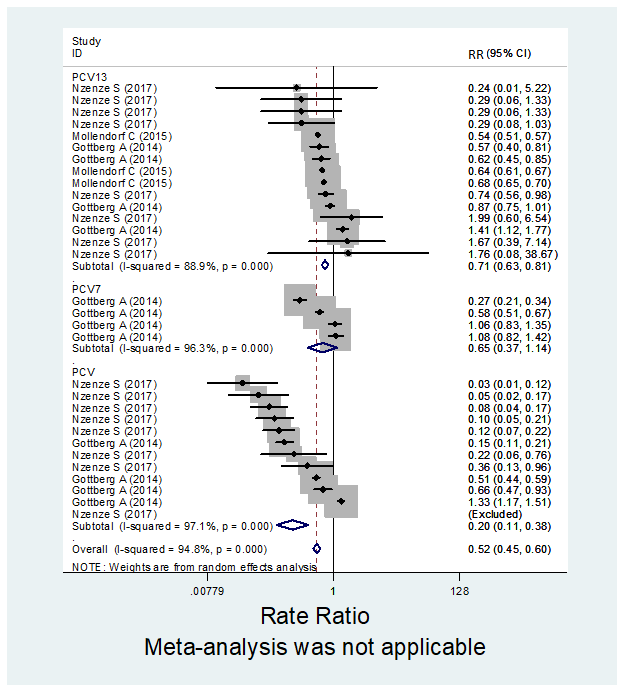
**

**Figure S11: Forest plot of the Effect of PCV against IPD among HIV-infected Children in case-control studies according to vaccine valency**

**
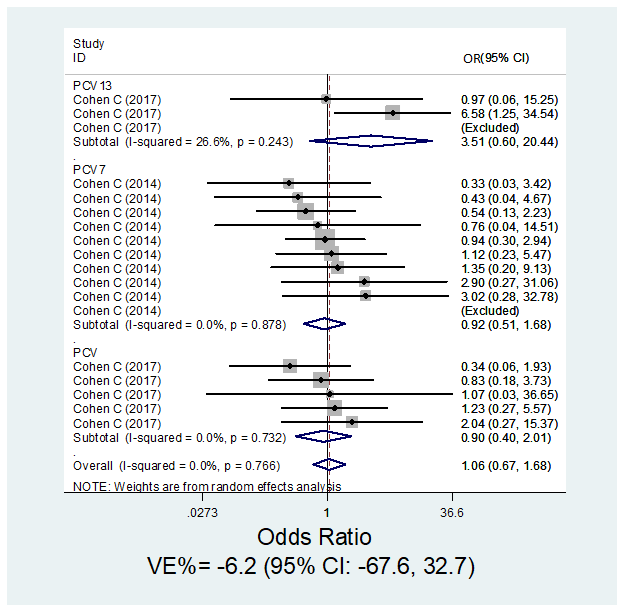
**

**Figure S12: Forest plot of the Vaccine effectiveness among HIV-uninfected Children in Case-Control studies according to vaccine valency**

**
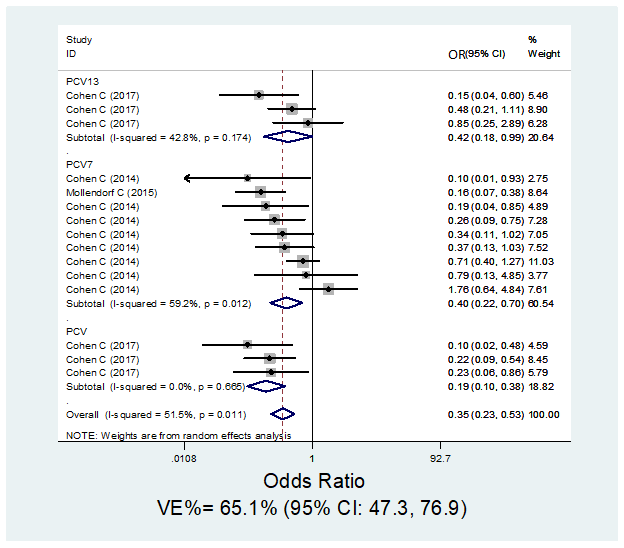
**

**Figure S13: Influential observation analysis (HIV-infected Children, pre-post studies)**

**Figure S14: Influential observation analysis (HIV-uninfected Children, pre=post studies)**

**Figure S15: Influential observation analysis (HIV-infected Children, case-control studies)**

**Figure S16: Influential observation analysis (HIV-uninfected Children, case-control studies)**

**Figure S17: Influential observation analysis (HIV-infected Children, randomized trials)**

**Figure S18: Influential observation analysis (HIV-uninfected Children, randomized trials)**

**Figure S19: Assessment of Publication Bias in Clinical Trial Studies in HIV-infected Children**

**Figure S20: Assessment of Publication Bias in Clinical Trial Studies in HIV-uninfected Children**

**Figure S21: Assessment of Publication Bias in Case-Control Studies in HIV-uninfected Children**

**Figure S22: Assessment of Publication Bias in Case-Control Studies in HIV-infected Children**

**Figure S23: Assessment of Publication Bias in Pre-post Studies in HIV-uninfected Children**

**Figure S24: Assessment of Publication Bias in Pre-post Studies in HIV-infected Children**
